# Changes in respiratory infection trends during the COVID-19 pandemic in patients with haematologic malignancy

**DOI:** 10.1186/s12890-024-03071-0

**Published:** 2024-05-26

**Authors:** Jiwon Ryoo, Seok Chan Kim, Jongmin Lee

**Affiliations:** 1 Division of Pulmonary and Critical Care Medicine, Department of Internal Medicine, Bucheon St. Mary’s Hospital, College of Medicine, The Catholic University of Korea, Seoul, Republic of Korea; 2 Division of Pulmonary and Critical Care Medicine, Department of Internal Medicine, Seoul St. Mary’s Hospital, College of Medicine, The Catholic University of Korea, 222 Banpo-daero, Seocho-gu, Seoul, 06591 Republic of Korea

**Keywords:** COVID-19, Community-acquired pneumonia, Immune deficiency, Haematologic malignancy

## Abstract

**Background:**

The coronavirus disease 2019 (COVID-19) pandemic has changed respiratory infection patterns globally. However, its impact on community-acquired pneumonia (CAP) in high-risk patients with haematological malignancies (HM) is uncertain. We aimed to examine how community-acquired pneumonia aetiology in patients with haematological malignancies changed during the COVID-19 pandemic.

**Methods:**

This was a retrospective study that included 524 patients with haematological malignancies hospitalised with community-acquired pneumonia between March 2018 and February 2022. Patients who underwent bronchoscopy within 24 h of admission to identify community-acquired pneumonia aetiology were included. Data on patient characteristics, laboratory findings, and results of bronchioalveolar lavage fluid cultures and polymerase chain reaction tests were analysed and compared to identify changes and in-hospital mortality risk factors.

**Results:**

Patients were divided into the ‘pre-COVID-19 era’ (44.5%) and ‘COVID-19 era’ (55.5%) groups. The incidence of viral community-acquired pneumonia significantly decreased in the COVID-19 era, particularly for influenza A, parainfluenza, adenovirus, and rhinovirus (pre-COVID-19 era vs. COVID-19 era: 3.0% vs. 0.3%, *P* = 0.036; 6.5% vs. 0.7%, *P* = 0.001; 5.6% vs. 1.4%, *P* = 0.015; and 9.5% vs. 1.7%, *P* < 0.001, respectively), whereas that of bacterial, fungal, and unknown community-acquired pneumonia aetiologies remain unchanged. Higher Sequential Organ Failure Assessment scores and lower platelet counts correlated with in-hospital mortality after adjusting for potential confounding factors.

**Conclusions:**

In the COVID-19 era, the incidence of community-acquired pneumonia with viral aetiologies markedly decreased among patients with haematological malignancies, with no changes in the incidence of bacterial and fungal pneumonia. Further studies are required to evaluate the impact of COVID-19 on the prognosis of patients with haematological malignancies and community-acquired pneumonia.

**Supplementary Information:**

The online version contains supplementary material available at 10.1186/s12890-024-03071-0.

## Background

The coronavirus disease 2019 (COVID-19) pandemic led to significant changes in respiratory infection patterns. After its outbreak in December 2019, in Wuhan, China, COVID-19 spread rapidly worldwide. Governments globally have extensively advocated for a range of measures to curb the COVID-19 pandemic, such as wearing masks, education on hand hygiene, social distancing, and travel restrictions. The Korean government has implemented several measures since February 2020 to prevent COVID-19 outbreaks, including active epidemiological investigations, quarantine of patients suspected of having the disease, and extensive public lockdowns [1]. The implementation of these strategies not only resulted in a decrease in the spread of COVID-19 but also substantially reduced that of other respiratory infections. Previous studies have reported a reduction in seasonal influenza activity [2–4], leading to a subsequent decrease in the occurrence of community-acquired pneumonia and hospital as well as intensive care unit (ICU) admissions [5].

Owing to both the characteristics of their condition and antineoplastic therapies, patients with haematological malignancies (HM) are immunocompromised and at high risk of infectious complications. They have a high incidence of community-acquired pneumonia (CAP), leading to substantial morbidity and mortality within this population [6, 7]. Early identification of CAP aetiology in patients with HM is important; however, despite the use of a comprehensive diagnostic workup, the aetiology of pneumonia is often under identified [8, 9]. Previous studies have revealed that immunocompromised patients with pneumonia of unknown aetiology have increased mortality rates [10, 11]. Furthermore, due to their immunocompromised status, the aetiology of CAP in these patients presents distinct characteristics from that in the general population. Therefore, a thorough exploration of the changing patterns in the aetiology of CAP, specifically in patients with HM, is warranted. Moreover, the impact of the COVID-19 era on the changing patterns of CAP in patients with HM remains uncertain.

## Methods

### Patients

**Fig. 1:**
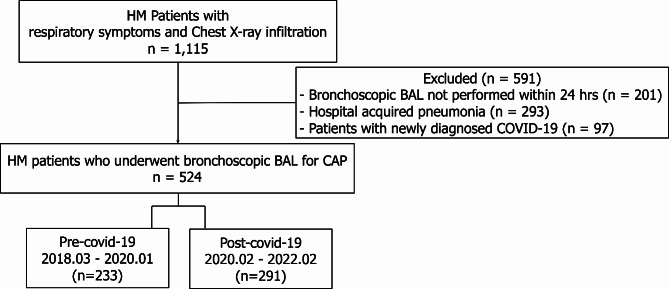
Study flow diagram. HM, haematologic malignancy; BAL, bronchoalveolar lavage; COVID-19, coronavirus disease 2019; CAP, community-acquired pneumonia

This study aimed to examine the changes in CAP aetiology in patients with HM during the COVID-19 pandemic. We retrospectively studied a cohort of patients with HM who visited the emergency department or outpatient clinic of Seoul St. Mary’s Hospital (Seoul, Korea) with respiratory symptoms and required admission between March 2018 and February 2022. In this hospital, more than 500 patients undergo haematopoietic stem cell transplantations annually. Eligible patients included those who exhibited abnormal findings on chest radiographs and underwent bronchoscopy (BRS) within 24 h of admission. Patients who did not undergo BRS within 24 h of admission, those diagnosed with hospital-acquired pneumonia (HAP), those diagnosed with a non-infectious disease, and those newly diagnosed with COVID-19 were excluded (Fig. 1). All patients admitted to the hospital from January 31, 2020, when COVID-19 testing became available in South Korea, were tested for COVID-19. Patients with COVID-19 were excluded because they could not undergo invasive procedures, such as bronchoalveolar lavage (BAL), to determine the aetiology of their respiratory infection, in accordance with hospital policy. Patients with HM were defined as those who underwent concurrent evaluations and treatment without remission [12]. This study was approved by the Institutional Review Board of Seoul St. Mary’s Hospital (KC23RISI0113), and the requirement for informed consent was waived by the Institutional Review Board of Seoul St. Mary’s Hospital.

### Data collection

Epidemiological and clinical data were collected from the patients’ medical charts at admission. Data included sex; age; laboratory findings, including white blood cell (WBC) count, absolute neutrophil count (ANC), absolute lymphocyte count (ALC), haemoglobin level, haematocrit level, platelet count, and C-reactive protein (CRP) level; characteristics of the HMs, including the type of malignancy, current status, and prior treatments; Sequential Organ Failure Assessment (SOFA) score at admission; body temperature at admission; and pattern of chest radiographic abnormalities. The results of BAL fluid culture, BAL galactomannan test, special staining (Gomori methenamine silver, periodic acid Schiff, or Ziehl–Neelsen) of BAL cells, atypical pneumonia serological panel (*Chlamydia, Legionella*, and *Mycoplasma*), blood culture, and serum galactomannan were also reviewed. A respiratory virus polymerase chain reaction (PCR) multiplex panel (AdvanSure RV real-time PCR Kit; LG Life Sciences, Seoul, Korea) was used to test for influenza A and B viruses, parainfluenza, respiratory syncytial virus (RSV), adenovirus, bocavirus, human metapneumovirus, coronavirus, and human rhinovirus in BAL samples. Nucleic acid extraction was performed using a QIAamp DNA Mini Kit in an automated extractor (Qiacube, Qiagen, Hilden, Germany).

Pneumonia was defined as the presence of a new infiltration on a chest radiograph at the time of the hospital visit with more than one of the following criteria: (1) new or increased cough with or without sputum production, (2) fever (temperature ≥ 38.0℃) or hypothermia (< 35.0℃), and (3) evidence of systemic inflammation (abnormal WBC count or increased CRP level) [6]. Invasive fungal diseases, including pulmonary aspergillosis, were defined based on the presence of compatible host factors, clinical features, and mycological evidence according to the European Organisation for Research and Treatment of Cancer/Mycoses Study Group criteria [13]. The diagnosis of *Pneumocystis jirovecii* pneumonia was based on the identification of the organism and/or positive PCR results from the BAL fluid, along with compatible clinical features and radiological findings [13]. CMV pneumonia was defined as cases that conformed to the criteria for either proven or probable CMV pneumonia and where the patient received appropriate treatment for CMV [14]. CAP cases of unknown aetiology were characterised by negative results in both BAL culture and PCR tests as well as negative serological examinations where no other identifiable cause of pulmonary infiltration could be found. Patients with HAP were defined as those admitted to the hospital for a reason other than acute respiratory infection, in whom respiratory symptoms developed ≥ 72 h after admission. Finally, we examined the clinical outcomes, including ICU admission and in-hospital mortality.

### Statistical analysis

Continuous variables were reported as median (range), whereas categorical variables were described as numbers (%). Patient characteristics were compared using the chi-squared test or Fisher’s exact test, as appropriate, for categorical variables and independent sample t-tests for continuous variables. The odds ratios (OR) and their corresponding confidence intervals (CI) were computed. Goodness-of-fit was computed to assess the relevance of the logistic regression model. All tests were two-sided, and a *P* value < 0.05 was considered statistically significant. All statistical analyses were performed using SPSS for Windows software (ver. 20.0; IBM Corp., Armonk, NY, USA) and R (ver. 4.3.1, R Foundation, Venna, Austria).

## Results

### Patient characteristics

Among the 1,296 patients with HM, respiratory symptoms, and chest radiographic infiltration admitted to our hospital between March 2018 and February 2022, 524 were included in our analysis (Fig. 1). As the Korean government implemented COVID-19 containment policies in February 2020, we divided the timeline into ‘pre-COVID-19’ and ‘COVID-19’ eras, using February 2020 as the point of demarcation [1]. The ‘pre-COVID-19 era’ and ‘COVID-19 era’ groups comprised 233 (44.5%) and 291 (55.5%) patients, respectively. Aside from a higher proportion of patients with bilateral pulmonary infiltration on their chest radiographs in the pre-COVID-19 era group than in the COVID-19 era group (81.1% vs. 69.4%, respectively, *P* = 0.003), no significant differences were observed between the two groups in terms of patient characteristics (Table 1).

**Table 1:** Baseline characteristics

Variables	Pre-COVID-19 era (*n* = 233)	COVID-19 era (*n* = 291)	*P* value
Age	57.0 (45.0–66.0)	60.0 (49.0–68.0)	0.054
Sex, male	136 (58.4)	181 (62.2)	0.423
Underlying haematologic malignancies			
Acute myeloid leukaemia	77 (33.0%)	101 (34.7%)	0.997
Acute lymphoblastic leukaemia	28 (12.0%)	35 (12.0%)	
Chronic myeloid leukaemia	11 (4.7%)	11 (3.8%)	
Multiple myeloma	26 (11.2%)	35 (12.0%)	
Myelodysplastic syndromes	36 (15.5%)	44 (15.1%)	
Lymphoma	29 (12.4%)	35 (12.0%)	
Others	26 (11.2%)	30 (10.3%)	
Disease status			
Active	169 (72.5)	217 (74.6)	0.670
Relapsed	49 (21.0)	77 (26.5)	0.179
HSCT recipients			
Autologous HSCT	14 (6.0)	30 (10.3)	0.108
Allogenic HSCT	91 (39.1)	123 (42.3)	0.513
SOFA score	3.0 (2.0–5.0)	3.0 (2.0–5.0)	0.233
Fever (temperature ≥ 38 °C)	158 (67.8%)	175 (60.1%)	0.085
Bilateral pulmonary infiltration on chest radiograph	189 (81.1)	202 (69.4)	0.003
Prognosis			
ICU admission	50 (21.5)	69 (23.7)	0.612
In-hospital mortality	43 (18.5)	63 (21.6)	0.427

Data are presented as a number (percentage) or median (interquartile range). HSCT, haematopoietic stem cell transplantation; SOFA, Sequential Organ Failure Assessment; ICU, intensive care unit

### Changes in CAP aetiology

**Fig. 2:**
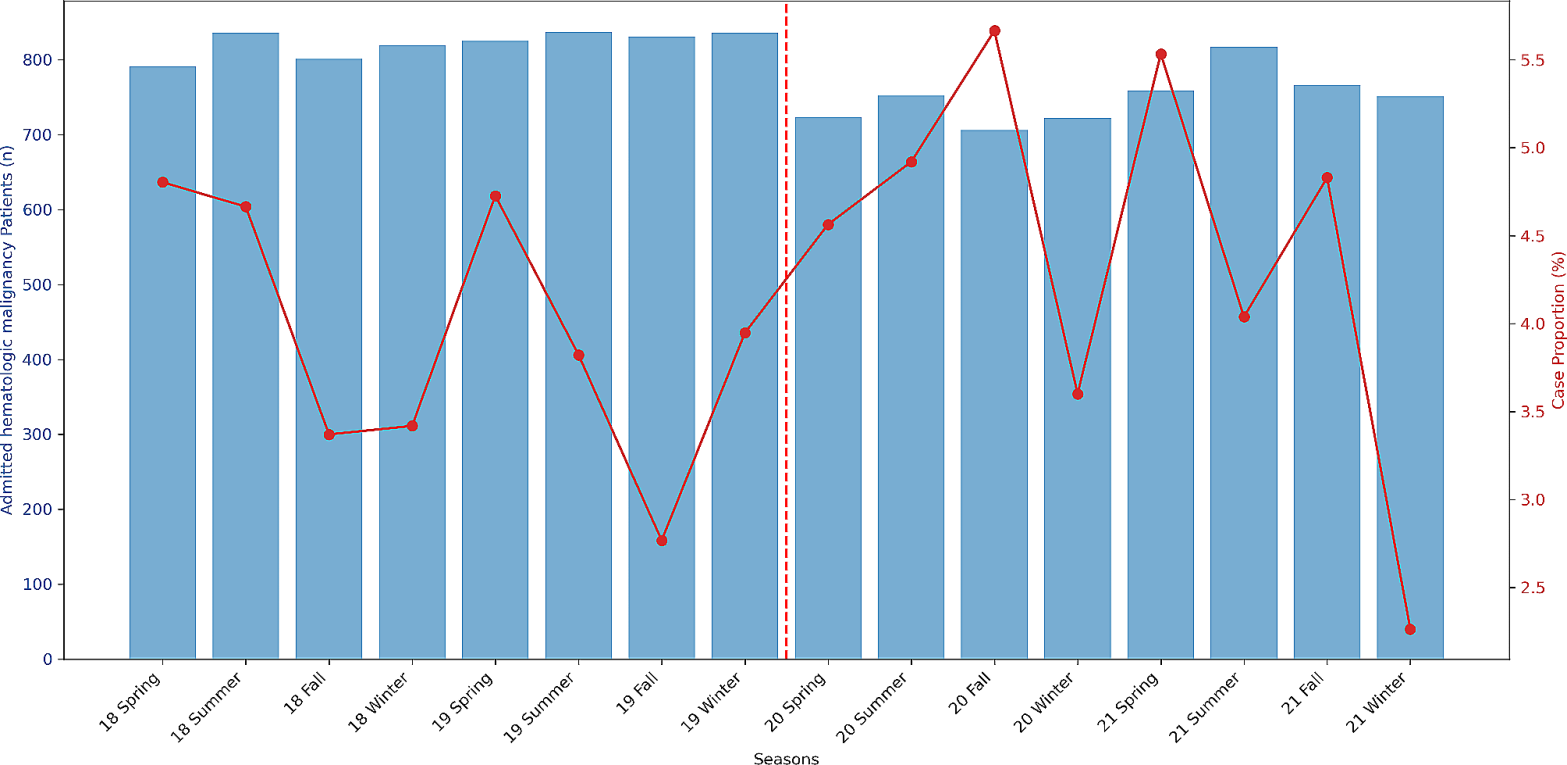
Number of hospitalized patients and the proportion of those with CAP from 2019 to 2021. Spring, from March to May; Summer, from June to August; Fall, from September to November; Winter, from December to February

**Fig. 3:**
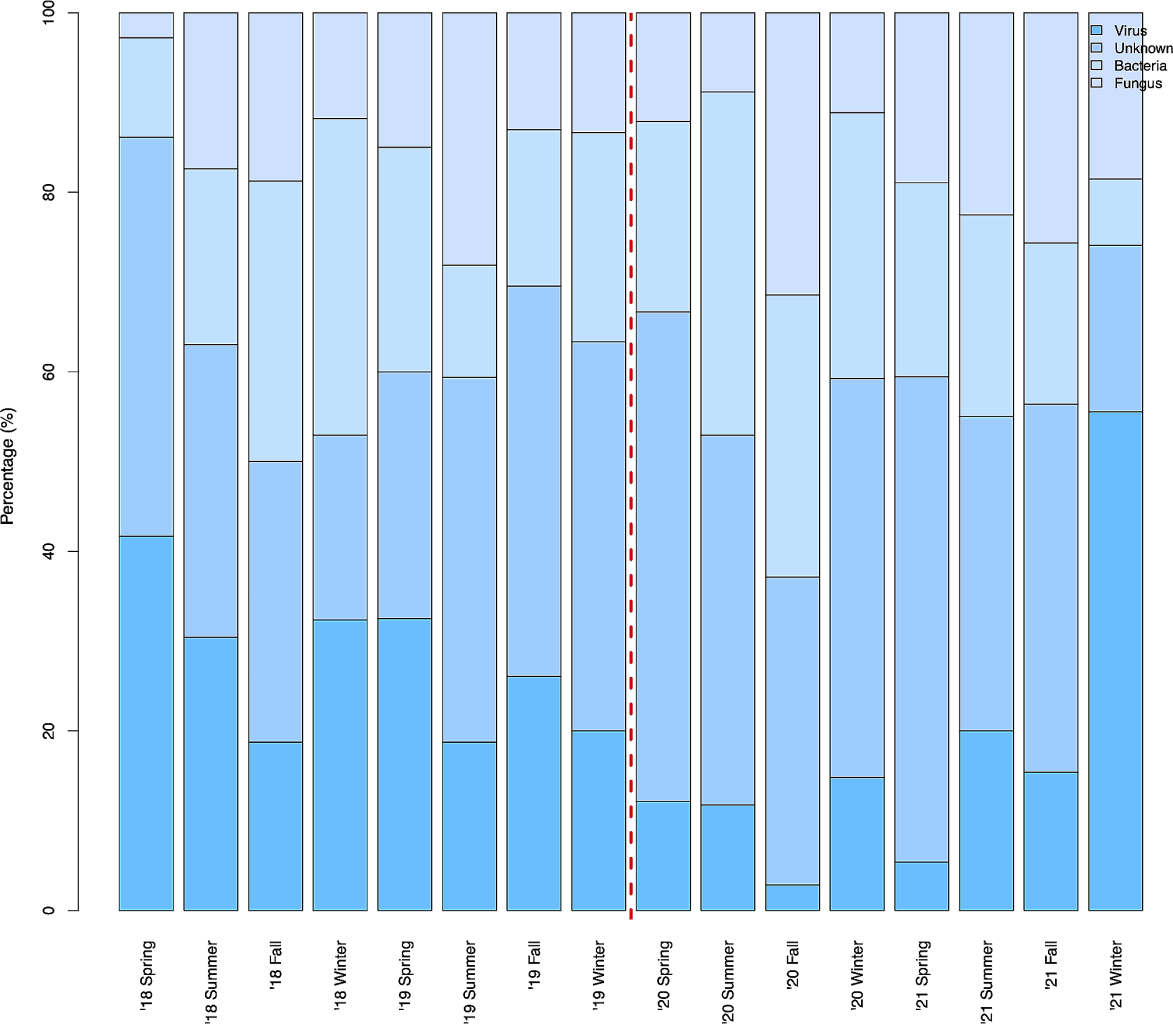
Change in CAP aetiology over time. Spring, from March to May; Summer, from June to August; Fall, from September to November; Winter, from December to February

**Fig. 4:**
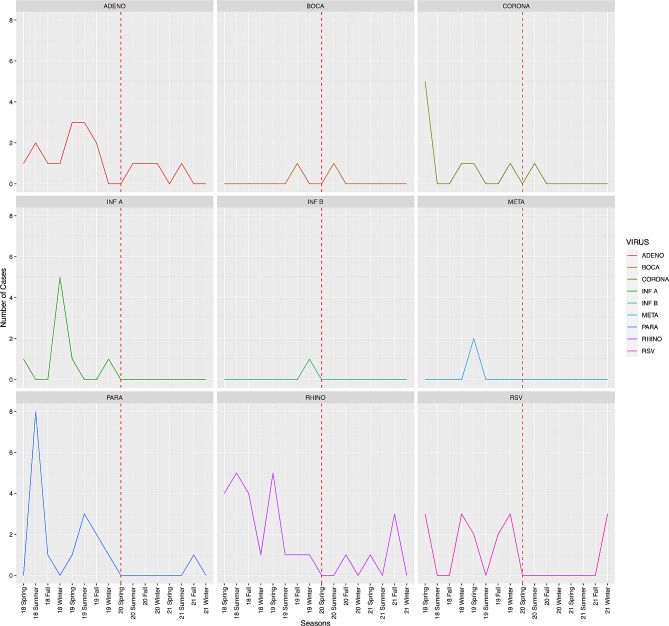
Seasonal distribution of respiratory virus infections. ADENO, adenovirus; BOCA, bocavirus; CORONA, human coronavirus; INF A, Influenza A; INF B, Influenza B; META, metapneumovirus; PARA, parainfluenza virus; RHINO, rhinovirus; RSV, respiratory syncytial virus. Spring, from March to May; Summer, from June to August; Fall, from September to November; Winter, from December to February

No significant differences were observed in the number of HM patients admitted or in the proportion of those admitted due to CAP when comparing the pre-COVID-19 era and COVID-19 era groups (*P* = 0.322) (Fig. 2). Table 2; Fig. 3 show the changes in the aetiology of CAP during the pandemic. During the pre-COVID-19 era, the proportions of aetiologies were as follows: unknown aetiology (39.5%), respiratory virus (30.7%), bacteria (23.2%), and fungus (16.3%). During the COVID-19 era, the proportion of aetiologies changed to the following: unknown aetiology (45.7%), bacteria (24.4%), fungus (18.9%), and respiratory virus (6.6%). From the pre-COVID-19 era to the COVID-19 era, the proportions of bacterial, fungal, and unknown aetiology CAP remained unchanged, whereas that of viral CAP significantly decreased. As shown in Table 2; Fig. 4, the proportion of respiratory viruses decreased during the pandemic. In particular, a significant reduction in the incidence of influenza A (pre-COVID-19 era vs. COVID-19 era: 3.0% vs. 0.3%, *P* = 0.036), parainfluenza (6.5% vs. 0.7%, *P* = 0.001), adenovirus (5.6% vs. 1.4%, *P* = 0.015), and rhinovirus (9.5% vs. 1.7%, *P* < 0.001) were observed during the pandemic. However, in the winter of 2021, an increase in the proportion of respiratory viruses was observed, which appeared to be primarily due to a rise in RSV cases (Figs. 3 and 4).

**Table 2:** Changes in pneumonia aetiology from the pre-COVID-19 era to the COVID-19 era

Pathogen species	Pre-COVID-19 era (*n* = 233)	COVID-19 era (*n* = 291)	*P* value
Bacteria	54 (23.2)	71 (24.4)	0.823
Gram-positive bacteria			
*Staphylococcus aureus*	4 (1.7)	8 (2.7)	0.623
*Streptococcus pneumoniae*	4 (1.7)	0 (0.0)	0.082
*Enterococci*	5 (2.1)	4 (1.4)	0.736
Gram-negative bacteria			
*Klebsiella pneumoniae*	5 (2.1)	11 (3.8)	0.409
*Escherichia coli*	1 (0.4)	5 (1.7)	0.335
*Pseudomonas aeruginosa*	9 (3.9)	11 (3.8)	1.000
*Chlamydia pneumoniae*	5 (2.1)	9 (3.1)	0.693
*Acinetobacter baumannii*	2 (0.9)	3 (1.0)	1.000
Mycoplasma pneumoniae	8 (3.4)	7 (2.4)	0.662
Mycobacterium tuberculosis	10 (4.3)	11 (3.8)	0.942
Others^*^	8 (2.8)	10 (3.4)	0.822
Fungus	38 (16.3)	55 (18.9)	0.512
Invasive aspergillosis	20 (8.6)	41 (14.1)	0.069
*Pneumocystis jirovecii*	18 (7.7)	20 (6.9)	0.838
Respiratory virus	71 (30.7)	19 (6.6)	< 0.001
Influenza A	7 (3.0)	1 (0.3)	0.036
Influenza B	1 (0.4)	0 (0.0)	0.913
Respiratory syncytial virus	10 (4.3)	6 (2.1)	0.227
Parainfluenza virus	15 (6.5)	2 (0.7)	0.001
Adenovirus	13 (5.6)	4 (1.4)	0.015
Metapneumovirus	2 (0.9)	0 (0.0)	0.386
Rhinovirus	22 (9.5)	5 (1.7)	< 0.001
Human coronavirus	7 (3.0)	2 (0.7)	0.093
Bocavirus	1 (0.4)	1 (0.3)	1.000
Diffuse alveolar haemorrhage	1 (0.4)	3 (1.0)	0.778
Cytomegalovirus	7 (3.0)	2 (0.7)	0.093
Unknown aetiology	92 (39.5)	133 (45.7)	0.180

Data are presented as number (percentage) or median (interquartile range). ^*^Others: Non-tuberculosis mycobacterium (4), *Legionella pneumoniae* (3), *Corynebacterium striatum* (3), *Enterobacter species* (2), *Achromobacter* species (1), *Staphylococcus haemolyticus* (1), *Moraxella catarrhalis* (1), *Stenotrophomonas maltophilia* (1), *Rothia mucilaginosa* (1), *Haemophilus influenzae* (1)

### Immune status and aetiology of CAP

An additional Table 1 illustrates the relationship between immune status of patients and the etiology of CAP [see additional Table S1]. The data indicates no significant overall variations in CAP etiology correlated with immune status. However, the percentage of patients with acute leukemia was lower among patients with respiratory viruses than those with other aetiologies (35.6% vs. 48.8%, *P* = 0.029). Additionally, a higher proportion of relapsed disease was noted in patients with CMV than in those with other aetiologies (77.8% vs. 23.1%, *P* = 0.001).

### Factors associated with increased mortality

An additional Table 1 presents comparisons of the clinical characteristics between patients experiencing in-hospital death and survivors [see additional Table S2]. Non-survivors had higher active HM rates (non-survivors vs. survivors: 83.0% vs. 71.3%, *P* = 0.020), CRP levels (12.2 [6.4–21.3] vs. 8.4 [3.7–17.5], *P* = 0.001), disease relapse rates (34.9% vs. 21.3%, *P* = 0.005), and SOFA scores (4.0 [2.0–5.0] vs. 3.0 [2.0–5.0], *P* < 0.001) and lower ANCs (1.4 [0.2–6.1] vs. 3.3 [0.6–6.4], *P* = 0.049), haemoglobin levels (8.6 [7.6–10,60] vs. 9.7 [8.4–11.8], *P* < 0.001), haematocrit levels (25.1 [22.1–31.2] vs. 29.3 [25.2–35.5], *P* < 0.001), and platelet counts (27.5 [14.0–73.0] vs. 98.0 [36.0–191.0], *P* < 0.001) than survivors.

The results of the logistic regression analysis of the clinical parameters used to evaluate the risk factors associated with in-hospital mortality are shown in Table 3. A high SOFA score and CRP level, an active disease, and a low haemoglobin level, haematocrit level, and platelet count were independent risk factors for in-hospital mortality. After adjusting for potential confounding factors, high SOFA scores and low platelet counts were independently associated with in-hospital mortality (*P* < 0.001 and *P* = 0.004, respectively).

**Table 3:** Logistic regression analysis results for in-hospital mortality

Variables	Univariable			Multivariable		
	Crude OR	95% CI	*P* value	Adjusted OR	95% CI	*P* value
SOFA score	1.42	1.29–1.57	< 0.001	1.28	1.15–1.44	< 0.001
Active disease	1.97	1.14–3.41	0.016			
Relapsed disease	1.98	1.25–3.15	0.004	1.62	0.99–2.68	0.057
COVID-19 era	1.22	0.79–1.88	0.366			
Absolute neutrophil count	1.00	0.97–1.03	0.946			
Haemoglobin level	0.84	0.77–0.92	< 0.001			
Haematocrit level	0.94	0.91–0.96	< 0.001			
Platelet count	0.99	0.99–0.99	< 0.001	0.99	0.99–1.00	0.004
C-reactive protein level	1.03	1.01–1.05	0.004			

OR: odds ratio; CI: confidence interval; SOFA: Sequential Organ Failure Assessment.

## Discussion

In the present study, we compared the aetiology of CAP among patients with HM before and during the COVID-19 pandemic. During the pandemic, numerous studies have focused on the incidence and treatment of COVID-19 in immunocompromised patients; however, limited research has been conducted on aetiologies of CAP other than COVID-19, particularly on changes in them, in such patients. Nonetheless, considering the susceptibility of immunocompromised patients to various opportunistic infections, it is crucial to examine the changes in pathogens that can cause CAP in the COVID-19 era.

In contrast to the findings of previous studies that reported a decrease in the incidence of CAP in the COVID-19 era [15–18], in this study, the number of patients with HM hospitalised with CAP in the COVID-19 era did not decrease compared with that during the pre-COVID-19 era. Notably, the incidence of viral pneumonia demonstrated a significant decrease; however, the incidence of bacterial and fungal pneumonia remained unaffected, in contrast to other research findings. Although previous studies have reported a decline in the incidence of bacterial pneumonia [15], this study has not exhibited any decrease in its incidence during the COVID-19 pandemic. The unchanged incidence of bacterial pneumonia despite the rigorous infection control measures adopted during the COVID-19 pandemic indicates that the most significant risk factor for bacterial CAP in patients with HM is their immune status [19].

The incidence of pneumonia caused by respiratory viruses, including influenza A, parainfluenza, adenovirus, and rhinovirus, decreased significantly during the COVID-19 pandemic. Bilateral pulmonary infiltration on chest radiographs also showed a significant decrease during the COVID-19 pandemic, which was hypothesised to be a result of a decreased incidence of viral pneumonia [20]. Respiratory viruses usually cause mild upper respiratory tract infections; however, previous studies have reported that respiratory viruses are important pathogens causing CAP in immunocompromised patients and older adults [21, 22]. As reported in other studies, the implementation of various preventive measures following COVID-19 outbreaks is likely to not only curtail the transmission of COVID-19 but also reduce the incidence of viral pneumonia caused by other respiratory viruses [2–4].

In our study, we observed an increase in RSV incidence during the winter of 2021, which appears closely linked to the RSV outbreak in South Korea, likely due to the relaxation of domestic infection control measures in November 2021 [23]. This increase suggests a ‘rebound’ effect in the transmission of respiratory viruses, particularly RSV, following the easing of public health measures implemented during the COVID-19 pandemic. The phenomenon could be attributed to the combination of reduced herd immunity during periods of decreased infection rates and the concurrent relaxation of these measures. This underscores the importance of continuous surveillance of RSV and other respiratory viruses following changes in public health policies, urging preparedness in the healthcare system for similar future scenarios.

CAP of unknown aetiology is relatively common among immunocompromised patients [24, 25]. In this study, a substantial number of patients (42.9%) presented with CAP of unknown aetiology, despite our only including patients who underwent BRS to minimise the number of patients with an undetermined aetiology. The incidence of CAP of unknown aetiology did not decrease during the COVID-19 pandemic; however, this may not necessarily indicate that these cases were not caused by pathogens such as viruses or bacteria. The lack of testing for atypical infections and diagnostic limitations of laboratory techniques prevents us from drawing a definitive conclusion. It is plausible that these cases were associated with the patients’ underlying diseases or immunocompromised status, but further investigation is needed to establish a causal relationship.

We investigated the risk factors for in-hospital mortality in patients with HM who had CAP. Consistent with previous studies [26, 27], high SOFA scores and low platelet counts were found to be risk factors for in-hospital mortality in patients with HM who had CAP. In general, while the CURB-65 (confusion, uraemia, respiratory rate, blood pressure, age ≥ 65 years) criteria or pneumonia severity index (PSI) are commonly used to predict the prognosis of patients with pneumonia, previous studies have shown that they are not effective in immunocompromised patients with cancer [28]. Thus, we opted to use the SOFA score instead. Thrombocytopenia is also associated with the severity of and poor prognosis in patients with infections [29, 30]. Platelets play a role in inflammation and host defence mechanisms against microbial agents. Additionally, thrombocytopenia reflects bone marrow failure in patients with HM [31]. Therefore, thrombocytopenia could be a significant predictor of poor prognosis in patients with HM and CAP.

This study has several limitations. First, its retrospective design and single-centre implementation may introduce a selection bias, potentially affecting the significance of our findings. Nevertheless, we meticulously examined all admitted patients with CAP who underwent BRS in this single, large, 4-year cohort with consistent treatment protocols. The objective of our study was to assess the impact of the COVID-19 pandemic on the changing pattern of aetiology of CAP in patients with HM; thus, the setting of this study did not significantly deviate from that of a prospective observational study. Second, conducting a single-centre study may have had limitations in determining the overall aetiology of CAP in all patients with HM. However, this institution serves as the largest Asian centre for haematological disorders, performing over 500 bone marrow transplantations annually. Therefore, these data can be considered reasonably representative. Third, patients’ COVID-19 histories were not investigated. Previous studies reported that COVID-19 is a risk factor for bacterial or fungal co-infections and can play a significant role in patient prognosis [32, 33]. However, due to the retrospective nature of the study, it was not possible to conduct an investigation. Moreover, COVID-19 presents unique challenges, including limitations on conducting bronchoscopy and comprehensive pathogen evaluations. This has inevitably impacted our study, as excluding COVID-19 cases may have skewed the overall assessment of the disease spectrum. Recognizing that we are now in the post-pandemic era, it becomes evident that including known respiratory virus infections, such as COVID-19, would offer a more meaningful insight. This inclusion would enable an analysis of the aetiology during different stages of the COVID-19 pandemic and assess its impact on mortality rates.

## Conclusions

In the context of pandemic eras such as that of COVID-19, understanding the changes in CAP aetiology among immunocompromised patients, including those with HM, is crucial. The CAP incidence in patients with HM did not decrease during the COVID-19 pandemic, unlike that in the general population. A notable shift in the aetiology of CAP emerged among patients with HM during the COVID-19 pandemic, with a significant reduction in the incidence of viral pneumonia but no change in that of bacterial and fungal pneumonia. Further research is needed to evaluate the effects of COVID-19 on the prognosis of patients with HM and CAP.

### Electronic supplementary material

Below is the link to the electronic supplementary material.

Supplementary Material 1

## Data Availability

The datasets used and/or analysed during the current study are available from the corresponding author on reasonable request.

## Abbreviations

ALC: Absolute lymphocyte count
ANC: Absolute neutrophil count
BAL: Bronchoalveolar lavage
BRS: Bronchoscopy
CAP: Community-acquired pneumonia
CI: Confidence intervals
CMV: Cytomegalovirus
COVID-19: Coronavirus disease 2019
CRP: C-reactive protein
HAP: Hospital-acquired pneumonia
HM: Haematological malignancies
ICU: Intensive care unit
OR: Odds ratios
PCR: Polymerase chain reaction
PSI: Pneumonia severity index
RSV: Respiratory syncytial virus
SOFA: Sequential Organ Failure Assessment
WBC: White blood cell
