# Supplementary material for: Changes in respiratory infection trends during the COVID-19 pandemic in patients with haematologic malignancy
Source: BMC Pulm Med. 2024 May 26;24:259. doi: 10.1186/s12890-024-03071-0 (PMC11129456; doi:10.1186/s12890-024-03071-0)
Supplement: Supplementary file 1 — Supplementary Material 1 [file 12890_2024_3071_MOESM1_ESM.docx]

Table S1. Correlation between immune status in patients and aetiology of pneumonia.

|  | Respiratory virus (n = 90) | Others (n = 428) | P value | Bacteria (n = 125) | Others (n = 399) | P value | Fungus (n = 93) | Others | P value | CMV (n = 9) | Others (n = 515) | P value | Unknown  etiology (n = 206) | Others (n = 318) | P value |
| --- | --- | --- | --- | --- | --- | --- | --- | --- | --- | --- | --- | --- | --- | --- | --- |
| Acute leukemia | 32 (35.6) | 209 (48.8) | 0.029 | 51 (40.8) | 191 (47.9) | 0.200 | 40 (43.0) | 202 (46.9) | 0.574 | 3 (33.3) | 239 (46.4) | 0.658 | 104 (50.5) | 138 (43.4) | 0.134 |
| Active disease | 63 (70.0) | 318 (74.3) | 0.478 | 90 (72.0) | 296 (74.2) | 0.713 | 71 (76.3) | 315 (73.1) | 0.605 | 9 (100.0) | 377 (73.2) | 0.153 | 160 (77.7) | 226 (71.1) | 0.115 |
| Relapsed disease | 28 (31.1) | 98 (22.9) | 0.130 | 24 (19.2) | 102 (25.6) | 0.183 | 25 (26.9) | 101 (23.4) | 0.567 | 7 (77.8) | 119 (23.1) | 0.001 | 53 (25.7) | 73 (23.0) | 0.535 |
| Allogenic HSCT | 45 (50.0) | 168 (39.3) | 0.077 | 48 (38.4) | 166 (41.6) | 0.595 | 35 (37.6) | 179 (41.5) | 0.564 | 4 (44.4) | 210 (40.8) | 1.000 | 77 (37.4) | 137 (43.1) | 0.228 |
| Autologous HSCT | 8 (8.9) | 35 (8.2) | 0.990 | 8 (6.4) | 36 (9.0) | 0.461 | 9 (9.7) | 35 (8.1) | 0.776 | 1 (11.1) | 43 (8.3) | 1.000 | 21 (10.2) | 23 (7.2) | 0.302 |
| ANC | 3.2  (0.9 - 6.1) | 2.9  (0.4 - 6.4) | 0.609 | 3.5  (0.7 - 8.6) | 2.9  (0.4 - 5.8) | 0.098 | 3.3  (0.5 - 6.3) | 3.0  (0.4 - 6.3) | 0.912 | 5.4  (1.9 - 5.9) | 3.0  (0.4 - 6.3) | 0.194 | 2.3  (0.4 - 5.8) | 3.4  (0.6 - 6.5) | 0.077 |

Data are presented as a number (percentage) or median (interquartile range). CMV, cytomegalovirus; HSCT, haematopoietic stem cell transplantation; ANC, absolute neutrophil count.

Table S2. Clinical factors affecting in-hospital mortality

| Variables | In-hospital  survivor  (n = 418) | In-hospital  non-survivor  (n = 106) | P value |
| --- | --- | --- | --- |
| Age | 59.0 (46.0 – 66.0) | 60.0 (50.0 – 68.0) | 0.189 |
| Sex, male | 248 (59.3) | 69 (65.1) | 0.331 |
| Disease status |  |  |  |
| Active | 298 (71.3) | 88 (83.0) | 0.020 |
| Relapsed | 89 (21.3) | 37 (34.9) | 0.005 |
| HSCT recipients |  |  |  |
| Autologous HSCT | 38 (9.1) | 6 (5.7) | 0.347 |
| Allogenic HSCT | 172 (41.1) | 42 (39.6) | 0.861 |
| SOFA score | 3.0 (2.0 – 5.0) | 4.0 (2.0 – 5.0) | < 0.001 |
| Fever (temperature ≥ 38°C) | 263 (62.9) | 70 (66.0) | 0.629 |
| Bilateral pulmonary infiltration on chest radiograph | 307 (73.4) | 84 (79.2) | 0.271 |
| Post-COVID-19 era | 228 (54.5) | 63 (59.4) | 0.427 |
| Aetiology of pneumonia |  |  |  |
| Bacteria | 97 (23.2) | 28 (26.4) | 0.572 |
| Fungus | 77 (18.4) | 16 (15.1) | 0.510 |
| Cytomegalovirus | 6 (1.4) | 3 (2.8) | 0.570 |
| Respiratory virus | 90 (21.5) | 16 (15.1) | 0.181 |
| Diffuse alveolar haemorrhage | 2 (0.5) | 2 (1.9) | 0.388 |
| Bronchiolitis obliterans | 6 (1.4) | 1 (0.9) | 1.000 |
| Cryptogenic organising pneumonia | 25 (6.0) | 1 (0.9) | 0.060 |
| Unknown aetiology | 157 (37.6) | 49 (46.2) | 0.128 |
| Laboratory findings |  |  |  |
| White blood cell count | 6.0 (2.4 – 10.8) | 5.6 (1.7 – 12.9) | 0.942 |
| Absolute neutrophil count | 3.3 (0.6 – 6.4) | 1.4 (0.2 – 6.1) | 0.049 |
| Absolute lymphocyte count | 1.0 (0.5 – 2.0) | 0.9 (0.5 – 2.0) | 0.722 |
| Haemoglobin level | 9.7 (8.4 – 11.8) | 8.6 (7.6 – 10.6) | < 0.001 |
| Haematocrit level | 29.3 (25.2 – 35.5) | 25.1 (22.1 – 31.2) | < 0.001 |
| Platelet count | 98.0 (36.0 – 191.0) | 27.5 (14.0 – 73.0) | < 0.001 |
| C-reactive protein level | 8.4 (3.7 – 17.5) | 12.2 (6.4 – 21.3) | 0.001 |

Data are presented as number (percentage) or median (interquartile range). SOFA, Sequential Organ Failure Assessment; HSCT, haematopoietic stem cell transplantation.
